# Supplementary figures and images for: Lobeglitazone inhibits LPS-induced NLRP3 inflammasome activation and inflammation in the liver
Source: PLoS One. 2023 Aug 24;18(8):e0290532. doi: 10.1371/journal.pone.0290532 (PMC10449201; doi:10.1371/journal.pone.0290532)

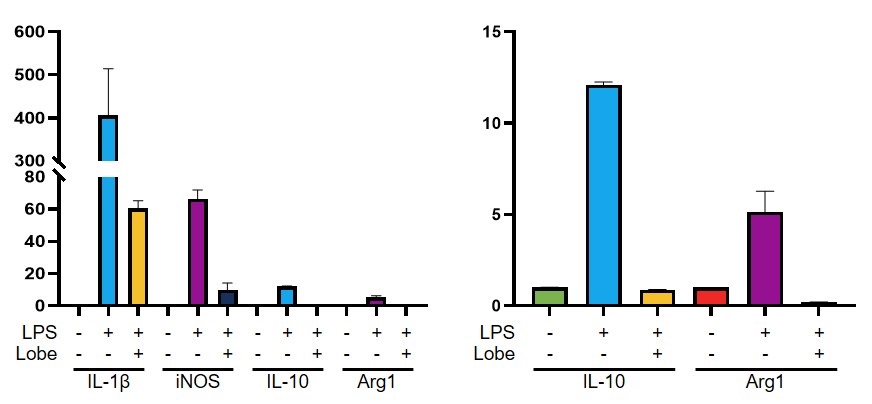

Supplement: S1 Fig — Representative real-time RT-PCR analysis of the expression levels of IL1β, iNOS, IL10, and Arg1. (JPG) [file pone.0290532.s001.jpg]

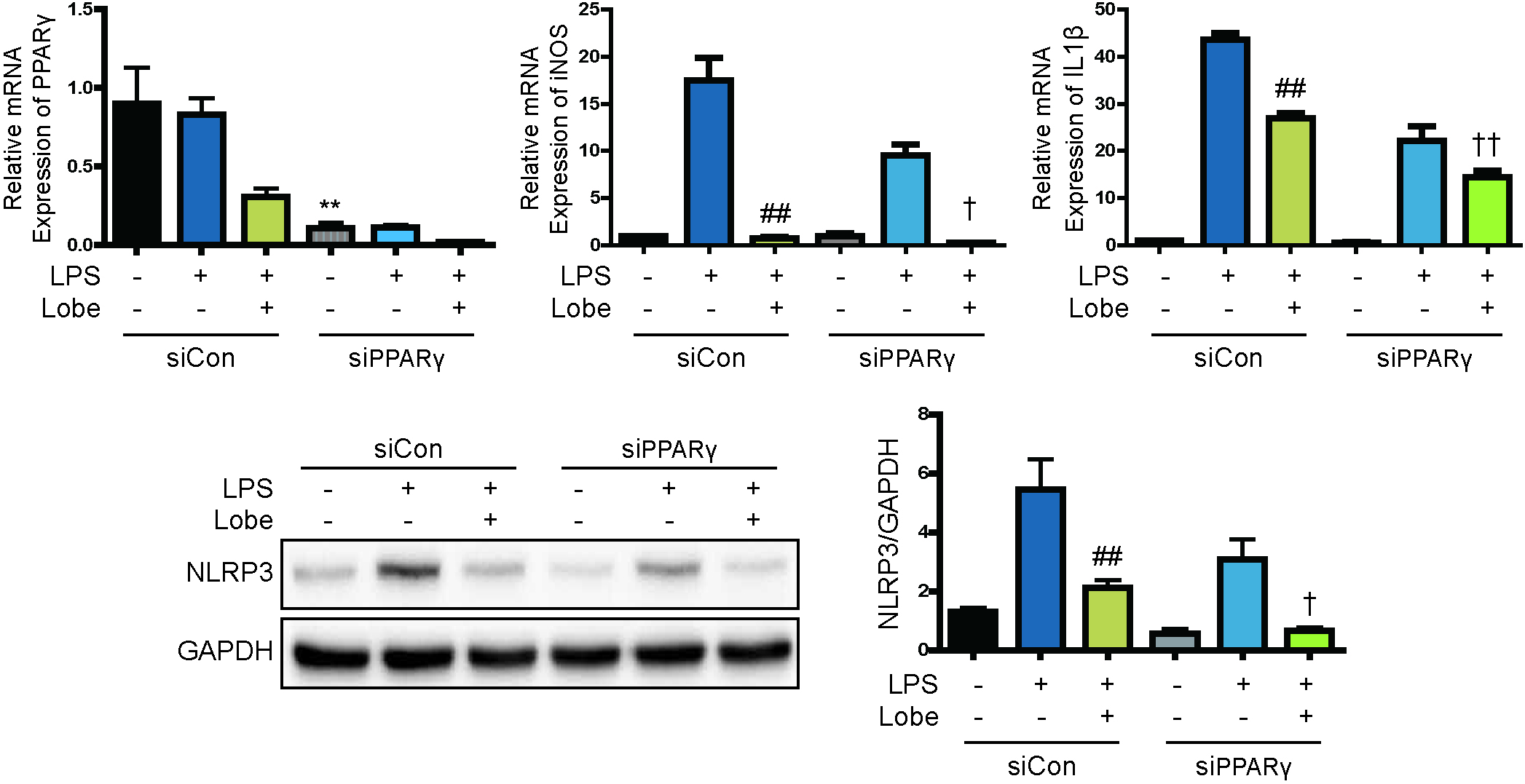

Supplement: S2 Fig — (A) Real-time RT-PCR analysis of the effect of PPARγ depletion on iNOS and IL1β expression. Primary KCs were transfected with 50 nM siPPARγ or control siRNA (siCon). *P < 0.05versus the control, #P < ##P < 0.01 versus the LPS-treated siCon, † P < 0.05, †† P < 0.01 versus the LPS-treated siPPARγ. (B) Western blot analysis of the effects of PPARγ depletion on NLRP3 expression. ##P < 0.01 versus the LPS-treated siCon, † P < 0.05 versus the LPS-treated siPPARγ. (JPG) [file pone.0290532.s002.jpg]

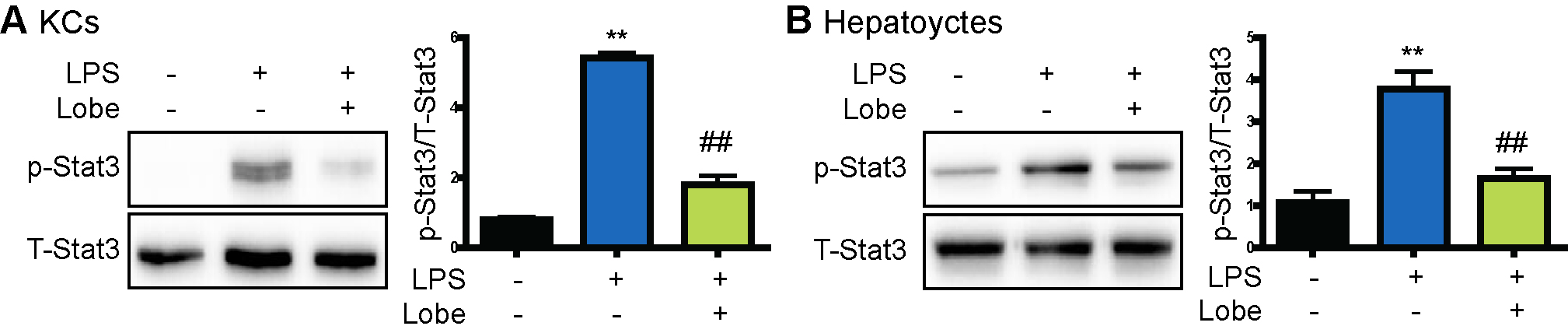

Supplement: S3 Fig — (A, B) Primary KCs and hepatocytes were pretreated with LPS for 2 h and then treated with lobeglitazone for 24 h. Western blot analysis of the effects of lobeglitazone on LPS-induced p-STAT3 expression. Data in the bar graphs are mean ± SEM. **P < 0.01 versus the control, ##P < 0.01 versus the LPS-treated group. (JPG) [file pone.0290532.s003.jpg]
