# Supplementary material for: Lobeglitazone inhibits LPS-induced NLRP3 inflammasome activation and inflammation in the liver
Source: PLoS One. 2023 Aug 24;18(8):e0290532. doi: 10.1371/journal.pone.0290532 (PMC10449201; doi:10.1371/journal.pone.0290532)

Fig 2.

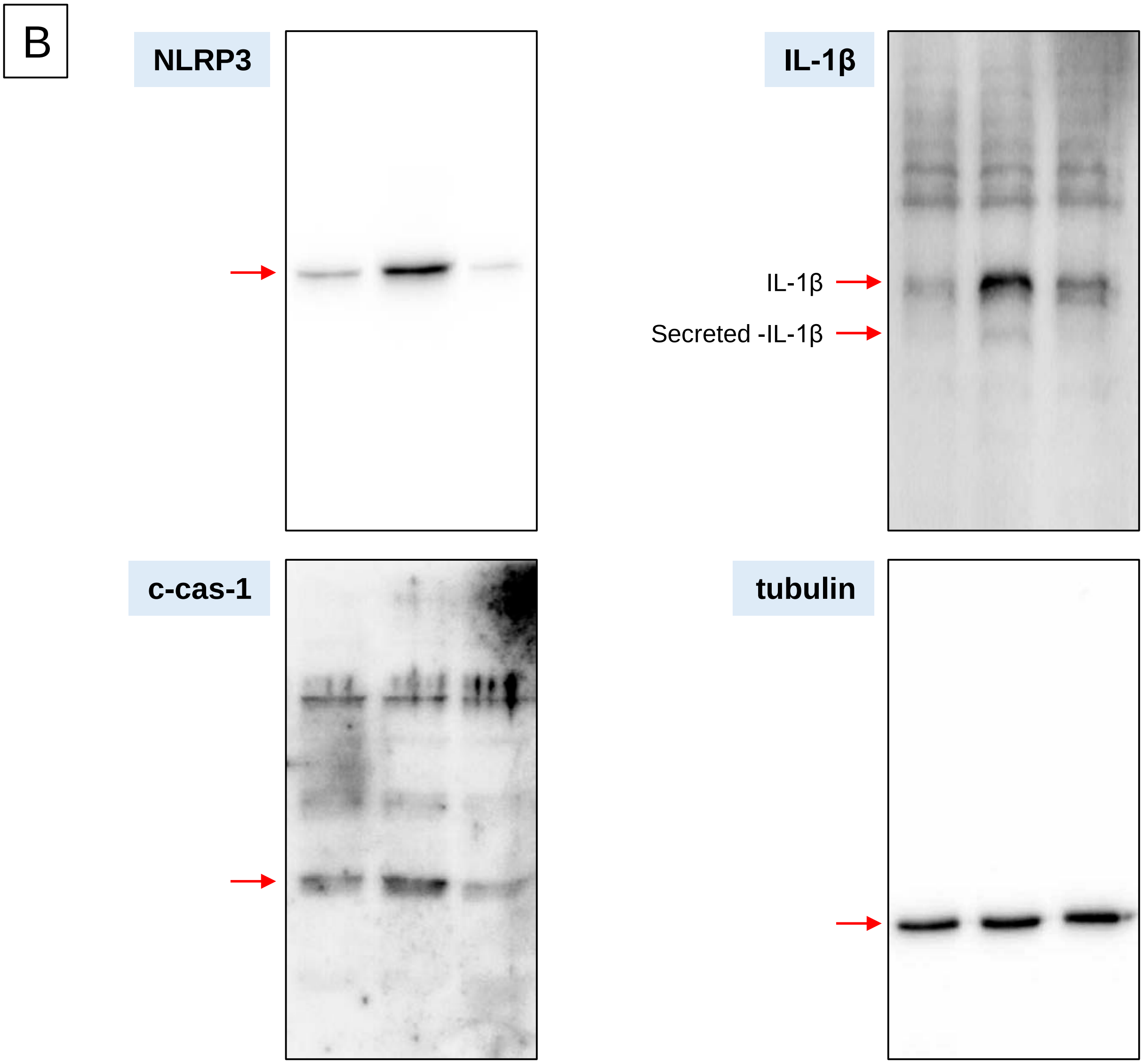

Fig 4.

C

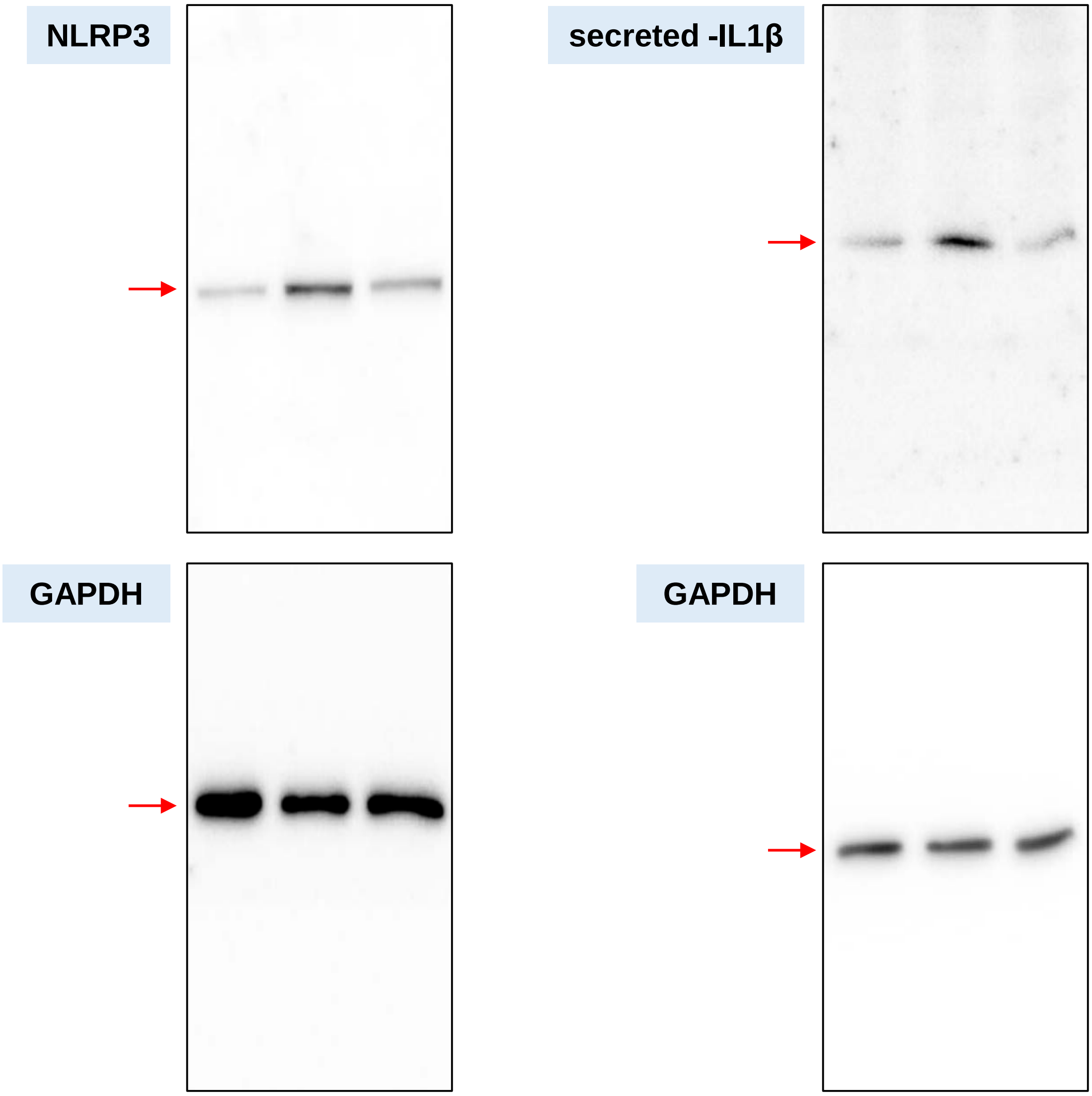

Fig 5.

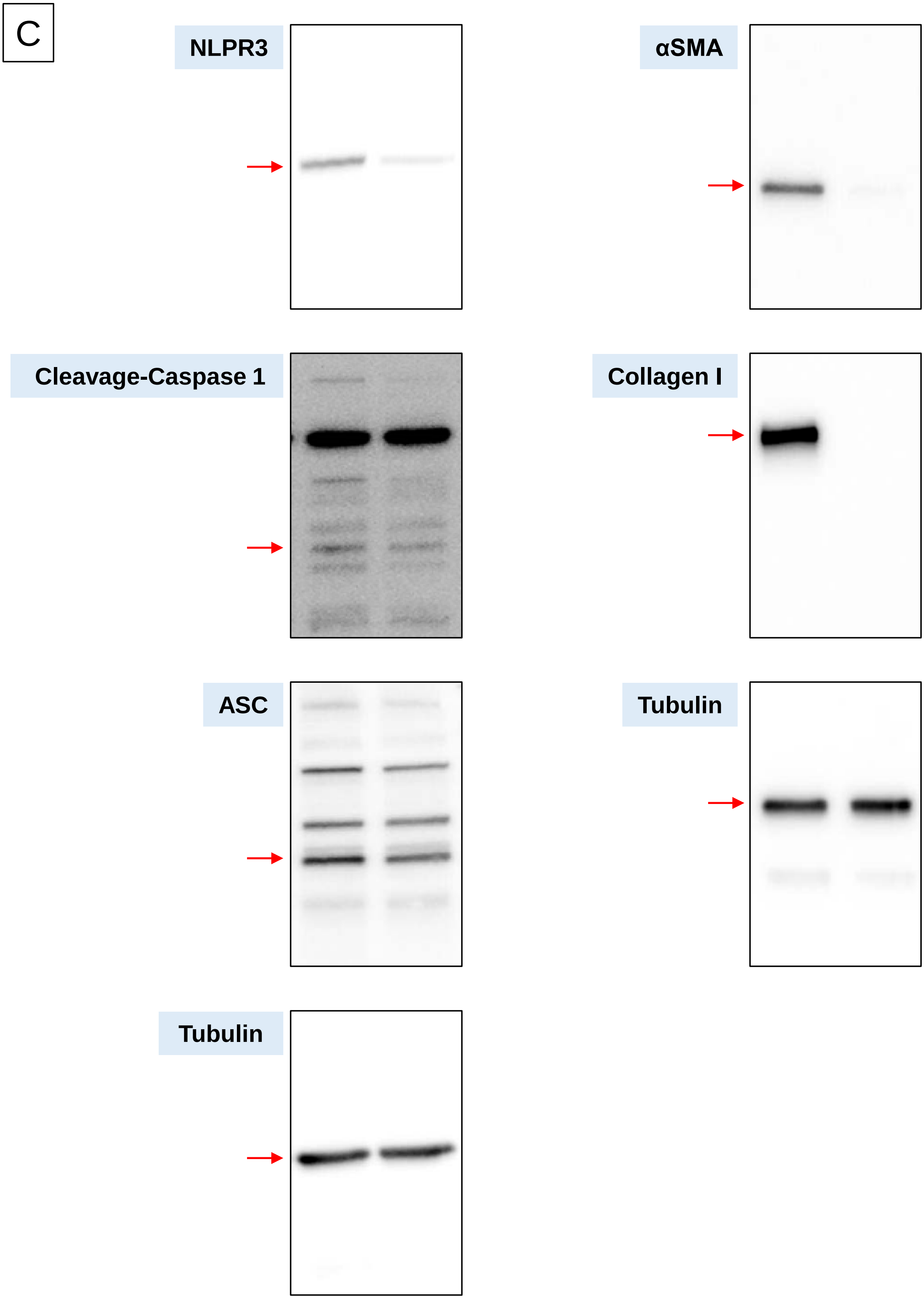
C
NLPR3
αSMA
Cleavage-Caspase 1
Collagen I
ASC
Tubulin
Tubulin

Fig 6.

B

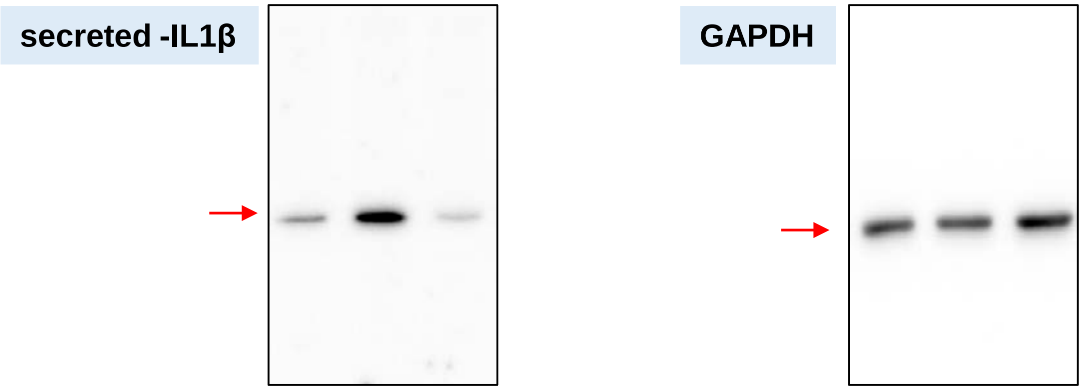

C

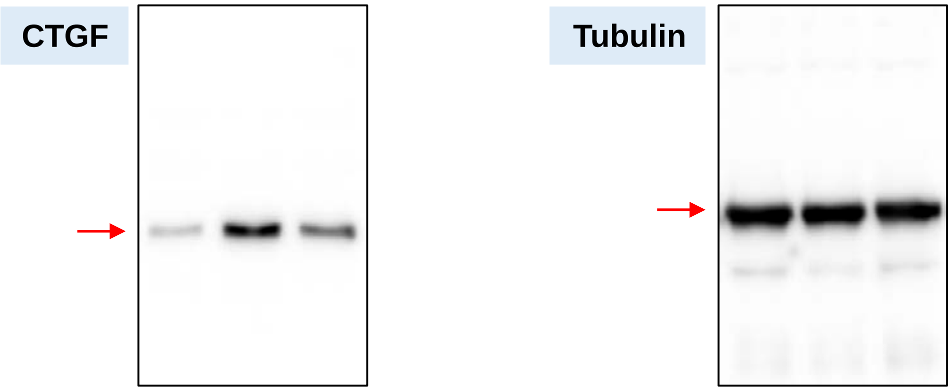

S Fig 1

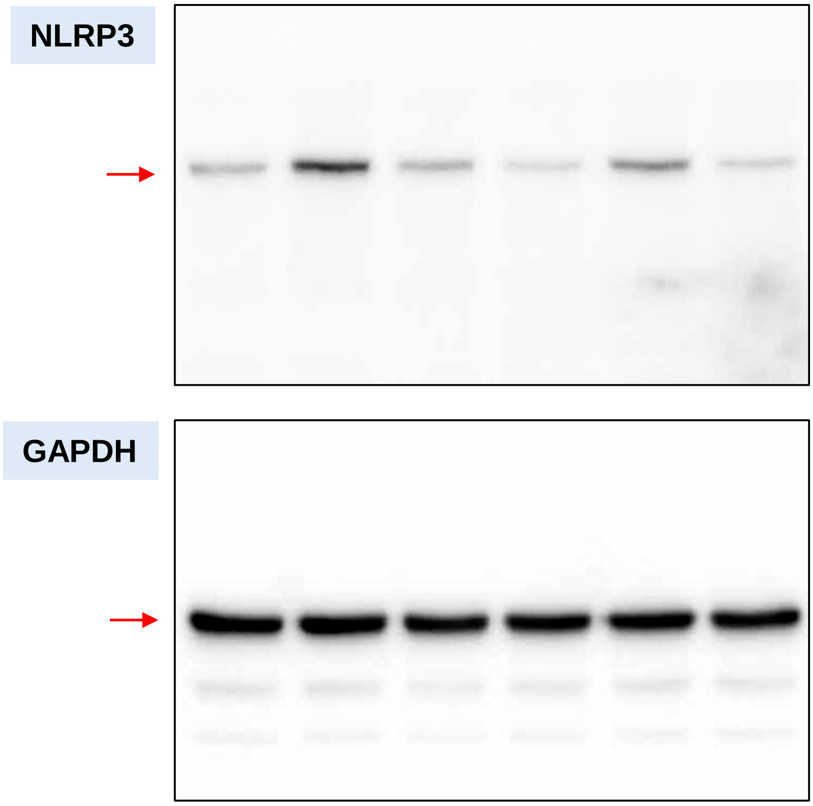

S Fig 2

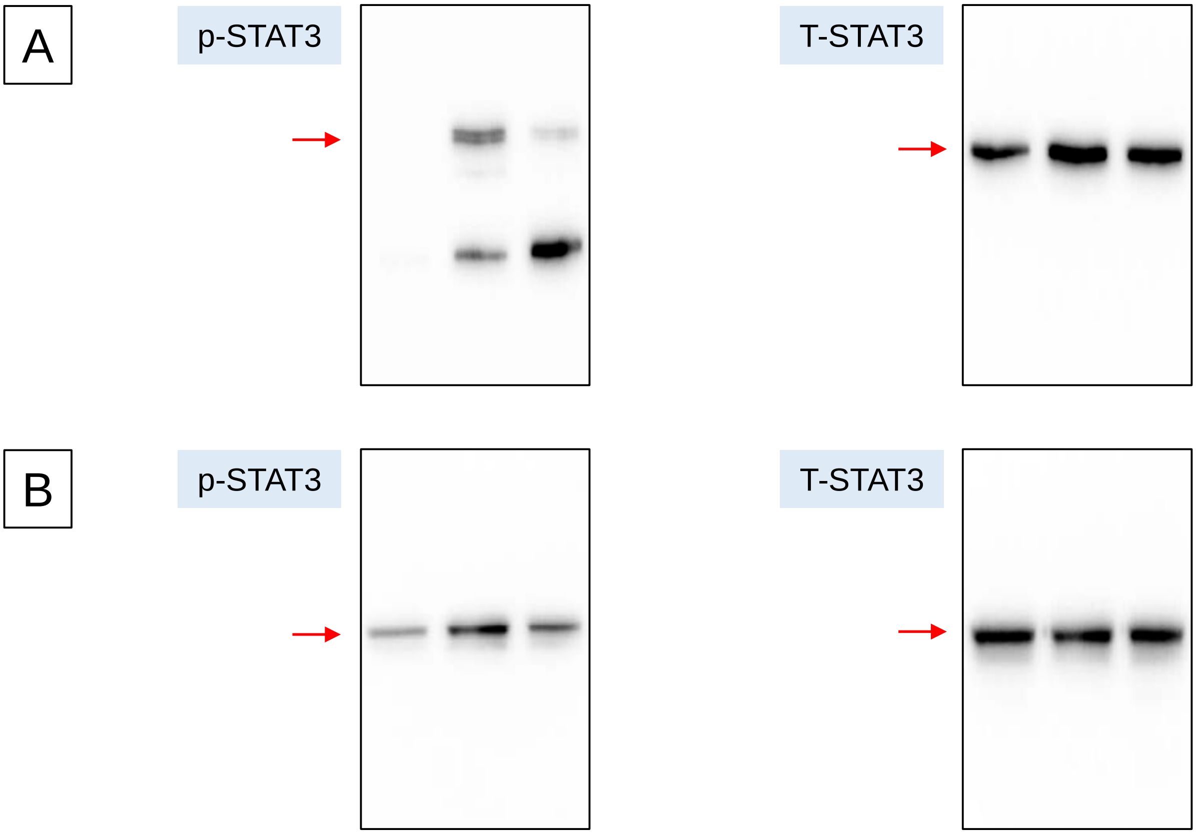

Supplement: S1 Raw images — (PDF) [file pone.0290532.s004.pdf]
